# Potential impacts of climate change on renewable energy in Egypt

**DOI:** 10.1007/s10661-024-12428-1

**Published:** 2024-02-14

**Authors:** Mahmoud Adel Hassaan, Mohamed Abdel Karim Aly Abdrabo, Hadeer Ahmed Hussein, Azza Abdallah Abdelhamid Ghanem, Hany Abdel-Latif

**Affiliations:** 1 Institute of Graduate Studies and Research, Alexandria University, 163 Horreya Avenue, Chatby, Alexandria, Egypt; 2 School of Management, Swansea University, Bay Campus, Fabian Way, Swansea, UK

**Keywords:** Renewable energy, Climate change, Egypt, Solar energy, Wind energy

## Abstract

The need for renewable energy sources is recently necessitated by attaining sustainability and climate change mitigation. Accordingly, the use of renewable energy sources has been growing rapidly during the last two decades. Yet, the potentials of renewable energy sources are generally influenced by several climatic factors that either determine the source of energy such as wind speed in the case of wind power or affect the performance of system such as the reduction in solar PV power production due to temperature increase. This highlights the need for assessing climate change impacts on renewable energy sources in the future to ensure their reliability and sustainability.

This paper is intended to assess impacts of climate change on wind and solar potential energy in Egypt by the year 2065 under RCP 8.5 scenario. For this purpose, a GIS-based methodology of three main steps was applied. The results revealed that solar energy potential in Egypt is expected to be relatively less vulnerable to climate change compared to wind energy. In this respect, it was found that while wind energy potential was estimated to range ± 12%. By the year 2065 under RCP 8.5 scenario, PV module power is expected to decrease by about 1.3% on average. Such assessment can assist in developing more sustainable and flexible renewable energy policy in Egypt.

## Introduction

Permanent economic and social development of electricity supply ensures sustainable any country (Didane et al., 2017). Accordingly, all countries seek to expand their installed electricity generation capacity to cope with increasing consumption of their rapidly growing population. For example, global renewable energy share of electricity capacity has expanded from 25.1 to 36.3% during the period 2011–2020 with an annual growth rate of about 5% (IRENA, 2021). The importance of renewable energy sources is highlighted under increasing interests on mitigating global warming and climate change and associated restriction on using fossil fuels to meet growing demand for electricity. This, therefore, highlights the need for switching to low-carbon energy sources such as wind energy that can play a crucial role in meeting increasing demand for electricity in the future. In this respect, the contribution of renewable energy of the total primary energy supply was recommended to reach two-thirds by 2050 (IRENA, 2018).

Such increasing importance of renewable energy sources entails exploring the future of these resources under climate change. Accordingly, potential impacts of climate change on wind energy were considered repeatedly. For example, some previous studies surveyed climate change impacts on renewable energy sources highlighting area of impacts, main trends, and gaps in this respect (Cronin et al., 2018; Pryor & Barthelmie, 2010; Solaun & Cerd, 2019). Others assessed the impacts of climate change on generation of renewable energy sources in different case studies worldwide (Duffy et al., 2014; Sawadogo et al., 2020; Spiridonov & Valcheva, 2017). More focused research work considered implications of climate change on potential wind generation (Davy et al., 2018; Miller & Keith, 2018; Tobin et al., 2014, 2016). Similarly, some studies focused on the vulnerability of wind energy to climate change-associated risks such as global warming and extreme weather events (Ohba, 2019; Pryor & Barthelmie, 2013).

In contrast, some previous research work examined the contribution of wind power turbines to global warming through redistributing heat through mixing the boundary layer. In this respect, it was argued that wind power would warm Continental US surface temperatures by 0.24 °C by 2100 (Miller & Keith, 2018). Nevertheless, climate benefits of wind power generation in terms of reducing GHG emissions are not comparable.

Among various climate change-associated risks, changing wind speed and increasing air temperature are expected to have direct impacts on wind energy. For example, it was suggested that due to cubed dependence of potential energy on wind speed (Ahmed, 2012; Al-Nassar et al., 2005; Didane et al., 2017; Grah et al., 2014), wind power density is very sensitive to changes in wind speed, i.e., any slight change in wind speed can have noteworthy impacts on the output of wind turbine (Davy et al., 2018; Ohba, 2019; Pryor & Barthelmie, 2010; Tobin et al., 2014). Moreover, it was argued that extreme weather events can reduce the electrical power production as turbines cannot operate under extremely high wind speed (Devis et al., 2018). Meanwhile, the expected increase in air temperatures will lead to slight decline in air density, which is one of the determinants of wind power density. This, in turn, may reduce electrical power generation (Pryor & Barthelmie, 2010, 2013). Indirectly, increasing diurnal and seasonal variations may lead to fluctuating electrical power production, thus can reduce the significance of wind as a reliable energy source (Carvalho et al., 2017; Pryor et al., 2020).

The global distribution of annual total irradiation and the annual energy generation potential reveal that while the tropical regions receive the highest annual irradiation, the largest energy potentials of more than 1800 kWh/kW PV are noticed in the Himalaya and Southern Andes regions, due to the combination of large irradiation values and low temperatures (Dubey et al., 2013). This highlights the impact of increasing temperature on reducing PV solar cell efficiency. Accordingly, it was suggested that expected temperature increase in the future under different climate change scenarios will contribute to reducing cell efficiency and energy output (ADB, 2012). In this context, PV power production in the Scandinavian countries is expected to decrease by the year 2050 about 10–12% due to climate change (Jerez et al., 2015).

As for climate change impacts on hydropower, it was argued that increased uncertainty and seasonality of precipitation pattern and consequent changes in water flows can affect power output of dams. Also, expected increase in surface evaporation associated with global warming can reduce water storage and power output (ADB, 2012).

In Egypt, the electricity consumption has been experiencing a rapid growth, where the per capita electricity consumption increased from 977 kWh in 2000 to 1600 kWh in 2019 with an annual increase rate of 3.4% on average (OECD/IEA, 2022). Such a trend of electricity consumption is expected to accelerate in the future motivated by population growth and climate change that may become an additional stressor leading to increasing need for colling and consequently electricity consumption due to temperature increase (Abdrabo et al., 2018).

**Fig. 1:**
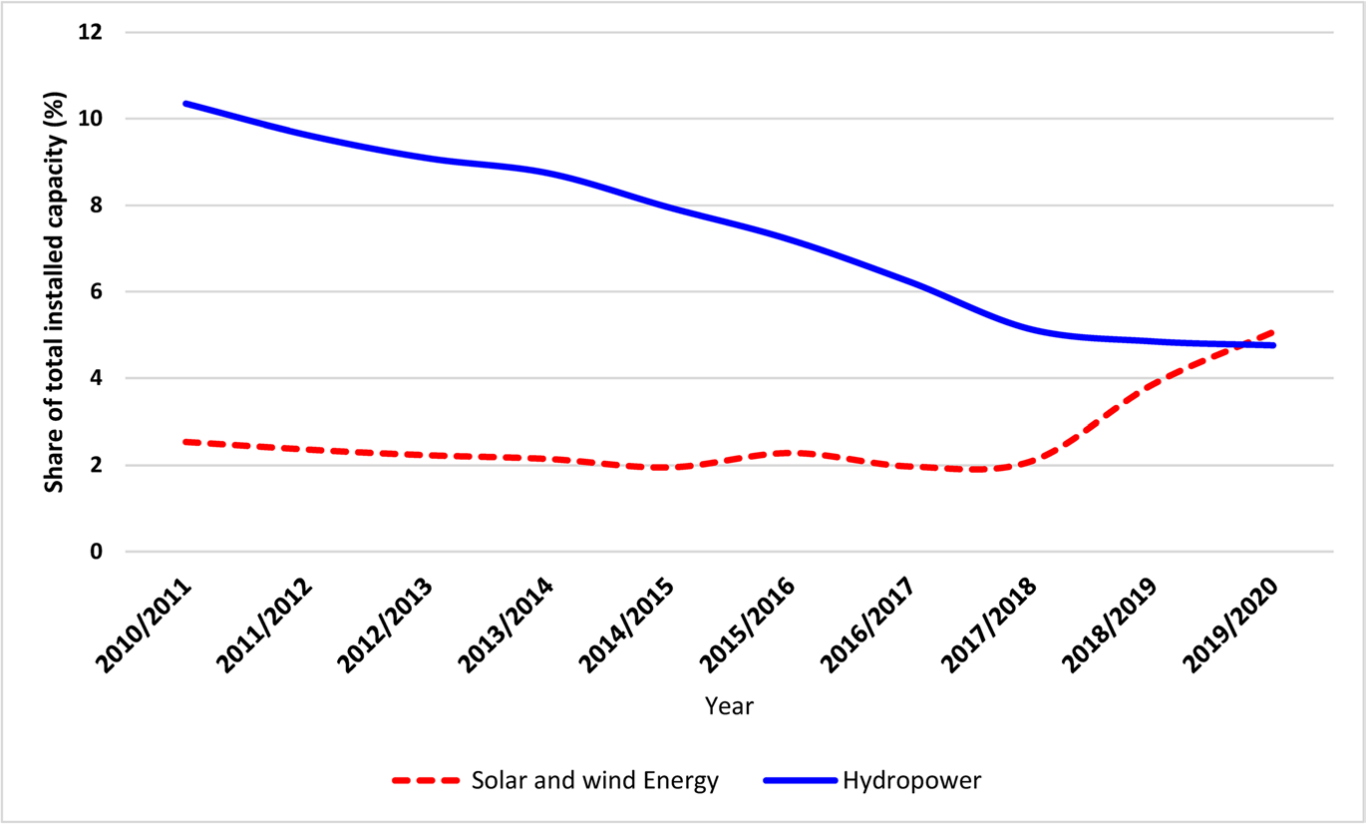
Share of renewable energy sources in total installed capacity of electricity in Egypt during the period 2011–2020

Meanwhile, installed electricity generation capacities have been increased from 27,049 to 59,530 M/W during the period 2010–2020 with an annual increase rate of 9.3%, on average. During the same period, installed electricity generation capacity of different renewable sources showed varied trends. In this respect, it was noted that the share of solar and wind energy sources in total installed capacity was doubled; the share of hydropower decreased from 10.4% in 2010 to 4.8% in 2020 (Fig. 1). Actually, such a noticeable decreasing share of hydropower in total installed electricity generation capacity in Egypt can be explained by unchanging installed capacity of hydropower electricity that are represented in only five hydropower plants that operate along the river Nile, from Aswan high dam to Assiut barrage (Eshra et al., 2021).

Up till 2015, the installed electricity generation capacities from renewable sources (solar and wind) were stable at 687 M/W representing about just 2% of the total installed electricity generation capacities. Henceforward, this proportion increased notably recording 5.1% of the total electricity generation capacities in 2020. This is mainly due to increased installed capacities of renewable energy sources during the period 2015–2020, which recorded on average an annual increase rate of 37.6% (EEHC, 2020). This trend highlights the growing importance of renewable energy source in electricity supply in Egypt.

Mostly, previous research work considering renewable energy in Egypt and Arab World either assessed the potential for wind power generation under current wind speed (Ahmed, 2010, 2012; Al-Dousari et al., 2019; Al-Nassar et al., 2005, 2019; Sebzali et al., 2013). Others conducted multi-criteria suitability analysis for siting solar farm (Hassaan et al., 2021; Alami Merrouni et al., 2018; Al Garni and Awasthi, 2017a;b; Dawod & Mandoer, 2016; El-Katiri & Husain, 2014; Effat, 2013; Gastli & Charabi, 2010). Moreover, several studies examined solar energy potential under current climate conditions (Awad et al., 2022; Nassar & Alsadi, 2019), while limited studies considered the hydropower potential (Eshra et al., 2021). These studies focused mainly on assessing potential of renewable energy under current conditions while no research was undertaken to examine climate change impact on renewable energy potentials in the future under climate change scenarios.

The paper in hand is intended to assess impacts of climate change on renewable energy sources in Egypt, particularly wind and solar energy, for the period 2050–2080, using 2065 as an indicator for this period under RCP 8.5 scenario. Such an assessment can contribute to filling the existing gap in wind energy research topic that was not addressed by previous studies. This was particularly found to be the case with only 4% of the total number of studies considering renewable energy focused on African case studies (Solaun & Cerd, 2019). The vulnerability of renewable energy sources to climate change impacts in Egypt was not considered by researchers in Egypt (Hassaan, 2018).

## Data and methodology

**Fig. 2:**
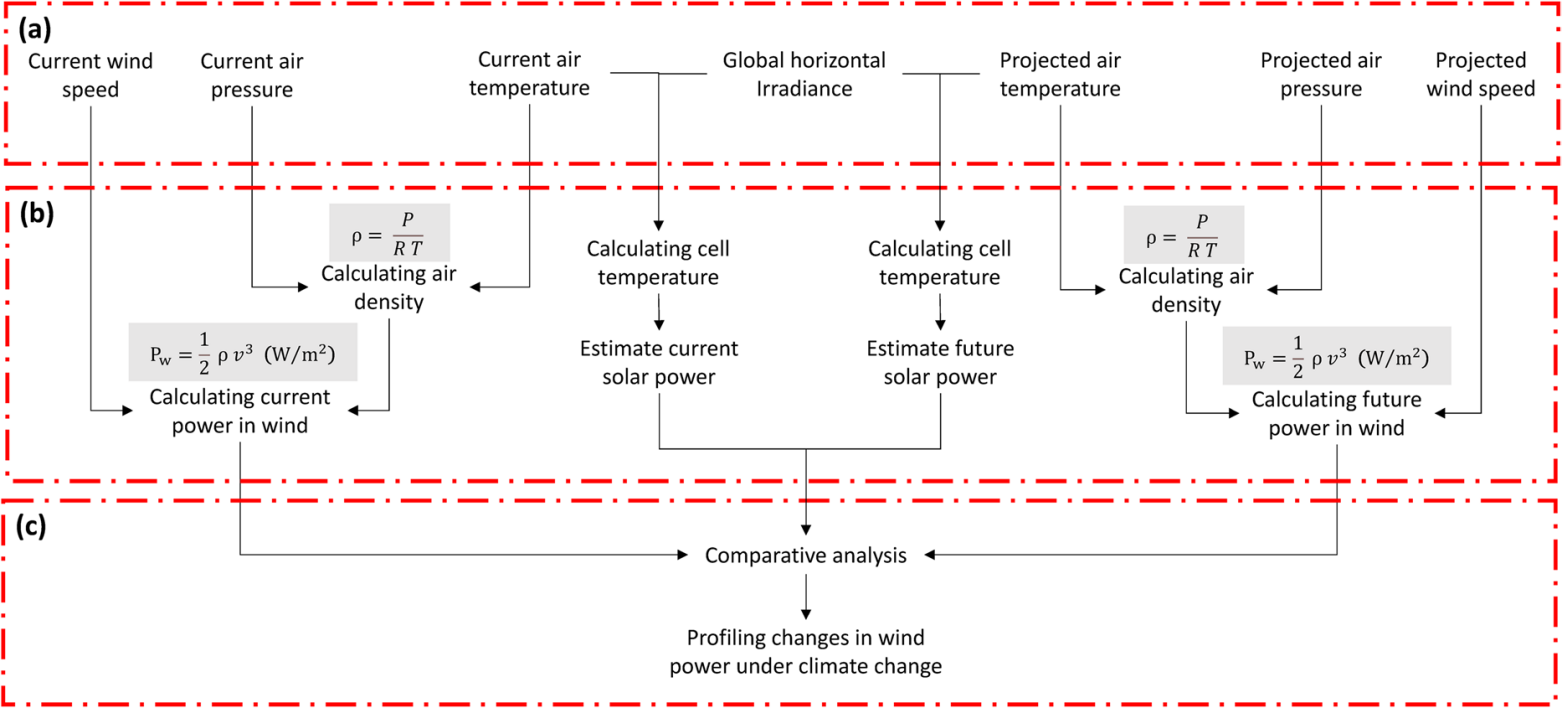
Methodology for assessing climate change impacts on renewable energy sources

Assessing climate change impacts on potential renewable energy sources, a methodology of three main steps was applied (Fig. 2) as follows:

- Data acquisition and manipulation: Data on various determinants of wind energy were acquired from Coordinated Regional Climate Downscaling Experiment (CORDEX) (CORDEX, 2020). The CORDEX provides ensembles of downscaled climate change scenarios using RCMs in time for the IPCC AR5 for different domains throughout the world using different dynamical techniques (Ebinger & Vergara, 2011; Nabipour et al., 2020). Downscaled historical data on wind speed, air temperature, and air pressure for reference period (1970–2005) as well as the projected data on the same variables under RCP 8.5 scenario over the period (2050–2080) with 25-km spatial resolution at monthly basis from CORDEX using RCA4 model were downloaded.

As per solar irradiation, data on global horizontal irradiation (GHI) in Egypt was collected from Global Solar Atlas (WBG, 2020), which provides data on GHI at national level in raster format with spatial resolution of 250 m.

(b)(b)(b) Estimating the potential of renewable energy generation: To estimate available energy in the wind under current as well as projected climatic conditions in the future, the following formula was employed (Tong, 2010):
  $$P_w=\frac12{\rho\;v}^3\;(\text{W}/\text{m}^2)$$

where:

*P*_*w*_: power in wind
*Ρ*: air density
*Ν*: wind speed m/s

Generally, air density is an important parameter in determining the power output of wind turbines (Ahmed, 2012; Nabipour et al., 2020; Sawadogo et al., 2020). The air density was calculated according to the following formula (Ahmed, 2010; Rehman & Al-Abbadi, 2007; Tong, 2010):

$$\rho = \frac{P}{R\;T} ({\text{kg}}/{{\text{m}}}^{3})$$

where:

*ρ*: air density
*R*: the gas constant = 287 J/kg K for air
*P*: air pressure
*T*: air temperature in Kelvin

Meanwhile, potential solar energy under current as well as projected temperature in the future can be estimated based on the negative relationship between the photovoltaic performance of PV modules and temperature due to the substantial decrease in the open-circuit voltage (*V*_oc_), where the power conversion efficiency of solar cells can be calculated according to the following equation (Green, 1982):

$$\eta =\frac{{J}_{{\text{sc}}} {V}_{{\text{oc}}} {\text{FF}}}{{P}_{{\text{in}}}}$$

where:

*J*_sc_: short circuit current density (mA/cm^2^)
*V*_oc_: open circuit voltage (V)
FF: fill factor (%)
*P*_in_: incident power (W/m^2^

Despite the increase of short circuit current density (*J*_sc_) as temperature increases, the *V*_oc_ decreases due to the rise of dark saturation current (*J*_*o*_) according to the following equation (Jäger et al., 2016):

$${V}_{{\text{oc}}}=\frac{kT}{q}\mathit{ln}\left(\frac{{J}_{L}}{{J}_{0}}\right)$$

where:

*K*: Boltzmann constant (Joule/Kelvin)
*T*: temperature (°C)
*q*: electric charge (coulomb)
*J*_*L*_: photo current (mA/cm^2^)
*J*_*o*_: dark saturation current (mA/cm^2^)

Since the decrease in voltage is faster than the increase in the *J*_sc_, the photovoltaic efficiency will decrease, and the power loss of PV module can be estimated by (Ross Jr, 1980):

$$\begin{array}{c}P= {P}_{{\text{STC}}}+{P}_{{\text{T}}-{\text{coeff}}}\times ({T}_{{\text{Cell}}}-25)^\circ {\text{C}}\\ {T}_{{\text{Cell}}}= {T}_{a}+(NOCT-20)\times \frac{G}{800 {~}^{W}\!\left/ \!{~}_{{m}^{2}}\right.}\end{array}$$

where:

*P*_STC_: power under standard test conditions
*P*_T-coeff_: power temperature coefficient
*T*_Cell_: solar cell temperature
*T*_*a*_: ambient temperature
NOCT: nominal operating cell temperature
*G*: irradiance power (W/m^2^)

(c)(c)(c) Profiling climate change impacts on the potential renewable power generation: This involved evaluating the potential wind and solar power generation by the year 2065 under RCP 8.5 scenario compared to current potential. Also, differences in potential wind and solar power were analyzed in order to delineate those areas with highest potentials in the future.

## Results and discussion

### Change in wind power

**Fig. 3:**
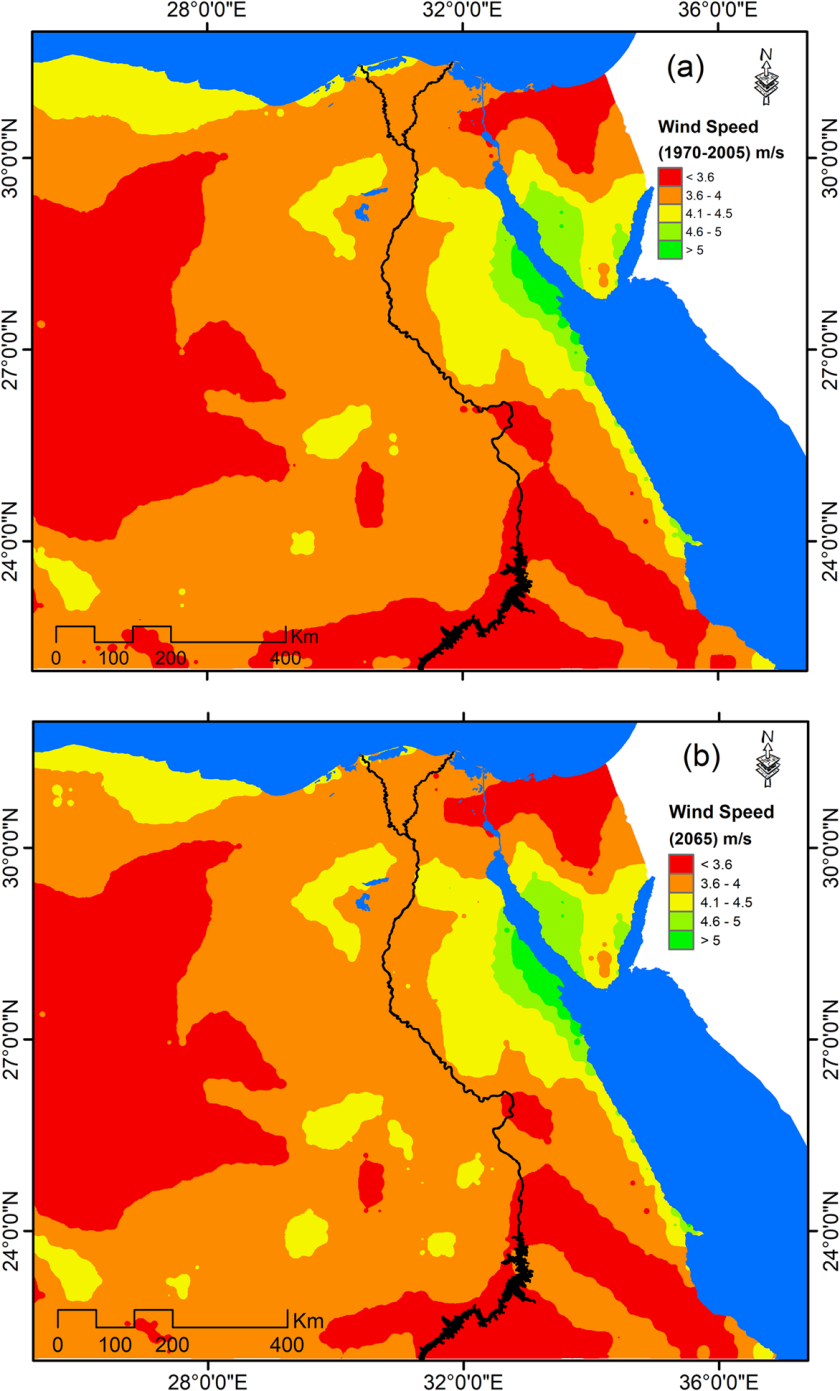
Annual average wind speed: **a** 1970–2005 and **b** 2065 under RCP 8.5

Generally, the annual average wind speed for the current climatic period (1970–2005) was found to range between 2.8 and 5.7 m/s. The lowest wind speed was recorded in northern Sinai, western desert, and southern parts of Egypt, where the wind speed does not exceed 3.5 m/s as most wind turbines usually start to generate energy at wind speed exceeding 3.5 m/s (Ahmed, 2012). This means that the suitable and viable sites with highest potentials for wind power generation are represented in Suez gulf, the Red Sea coastal strip, the northwestern coastal strip alongside Mediterranean in addition to some spots in the western desert, where the wind speed exceeds 4 m/s on average (Fig. 3a). Under RCP 8.5 scenario, there would be marginal difference in wind speed by the year 2065 (Fig. 3b).

**Fig. 4:**
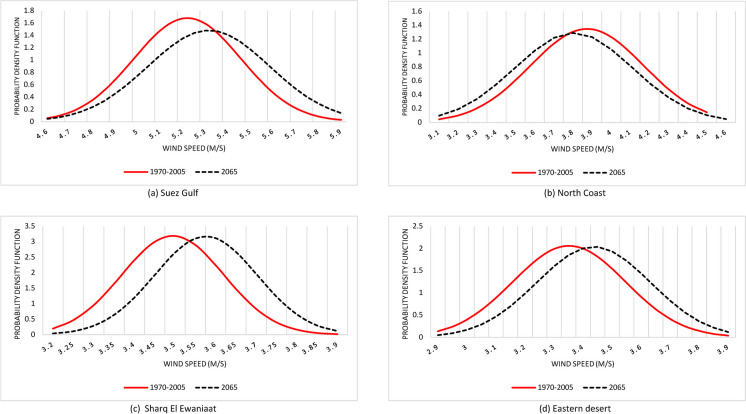
Gaussian distribution of projected wind speed by the year 2065 under RCP 8.5 scenario compared to 1970–2005

Generally, different parts of Egypt are expected to have varied trends of changes in wind speed. This is emphasized by Gaussian distribution projected wind speed by the year 2065 under RCP 8.5 scenario compared to 1970–2005, which revealed declining wind speed in the northern coastal zone (Fig. 4). This can be due to the expected rising temperatures and subsequent reduction in the thermal difference between polar regions and the tropics, which will lead to decrease in mean mid-latitude wind speeds (Ebinger & Vergara, 2011).

In contrary, other parts of Egypt are expected to experience a relatively increasing wind speed. Despite such an increasing trend, the wind speed in those parts, except for Suez Gulf, is expected to be below 4 m/s, which means low potentials for wind energy. Furthermore, the expected increase in wind speed is noticed to be slight.

**Fig. 5:**
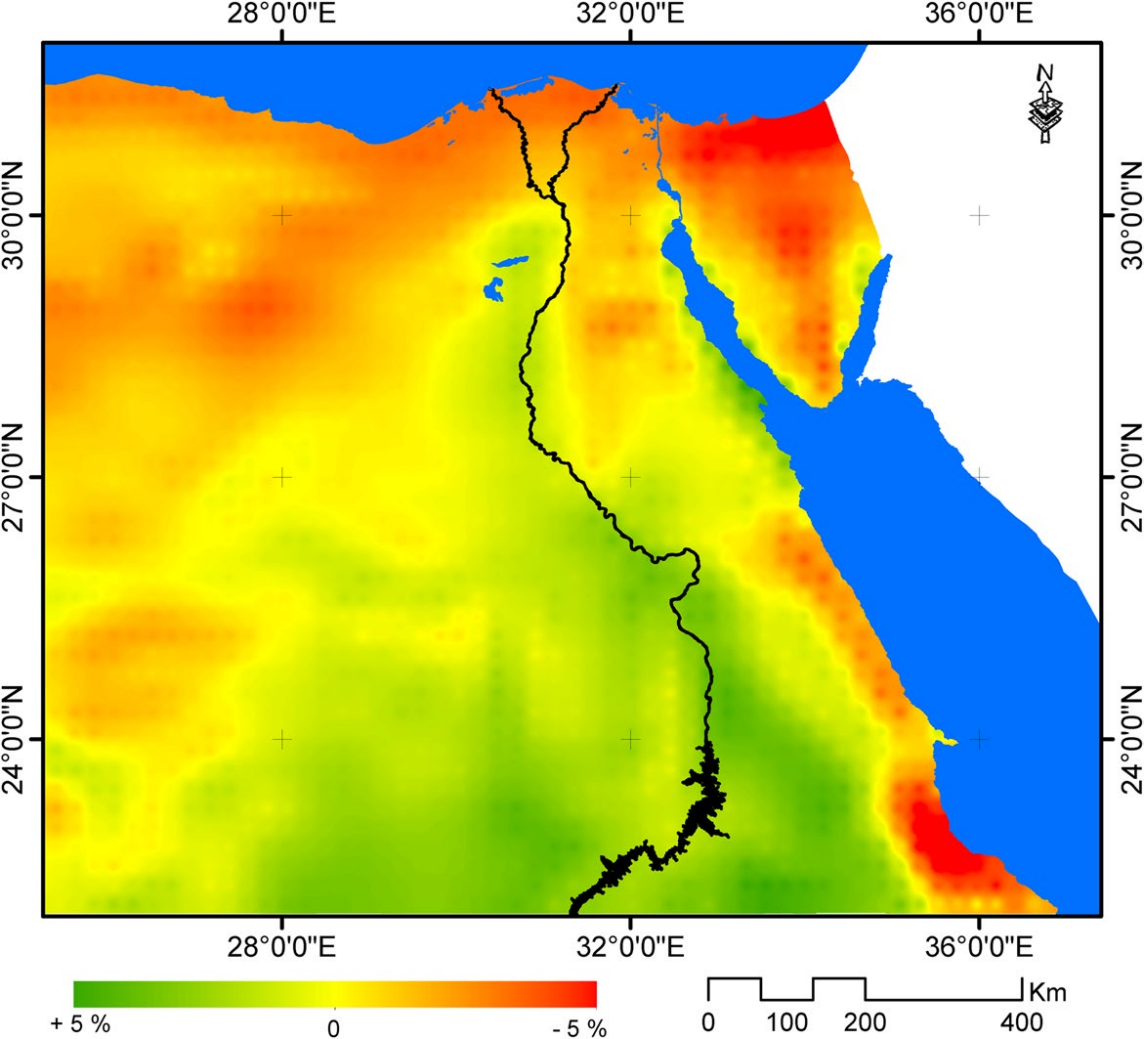
Relative change in wind speed by the year 2065 under RCP 8.5 compared to 1970–2005

In relative term, the change in wind speed will be within ± 5% compared to current climatic conditions. The highest reduction rate in wind speed is expected noted in the Red Sea coastal strip and northern coastal alongside the Mediterranean. Meanwhile, the highest increase rate is expected to be in Suez Gulf, Nile Valley, Sharq El Ewainatt, and southern parts of the eastern desert (Fig. 5).

**Fig. 6:**
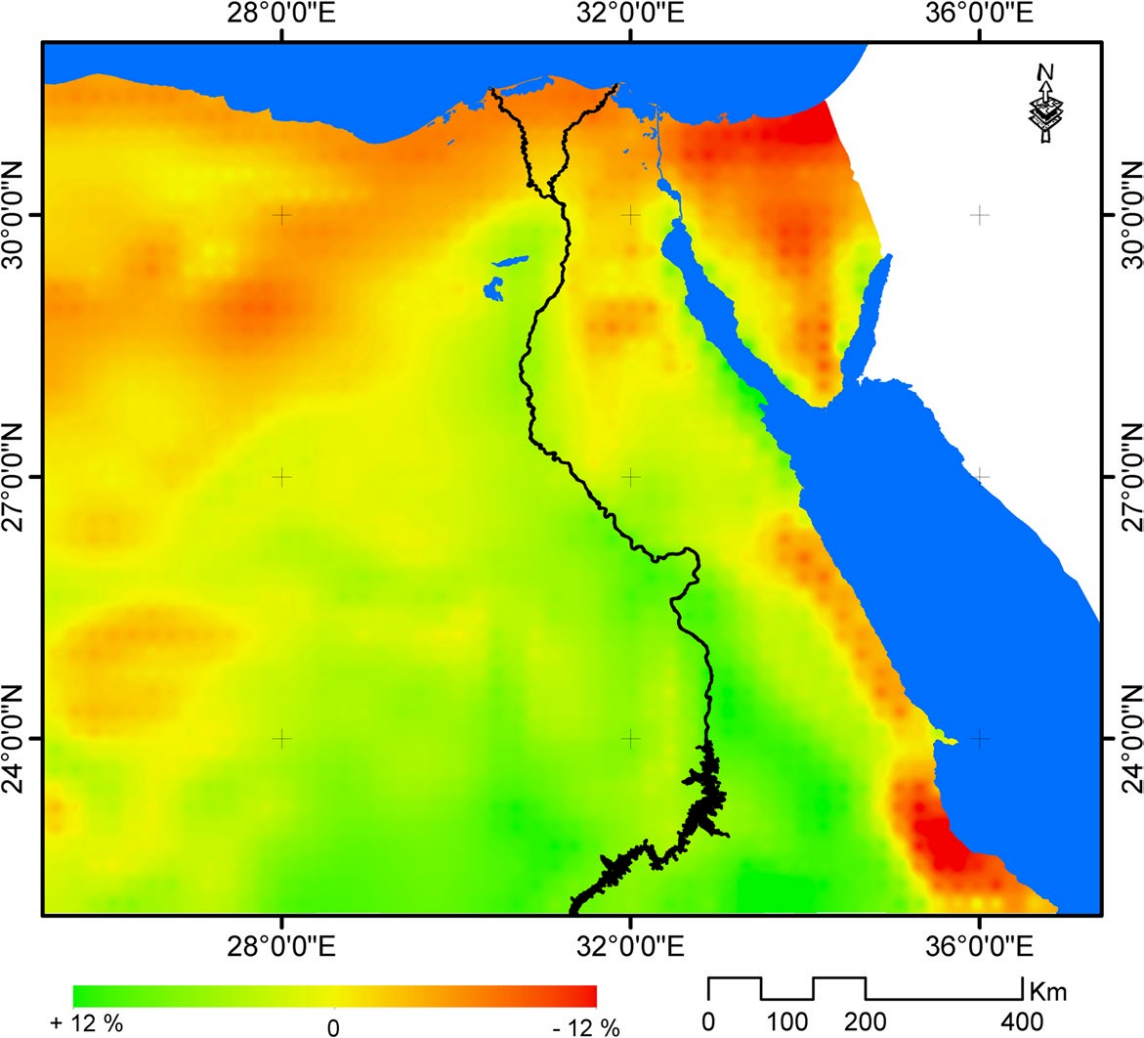
Relative change in wind energy potential by the year 2065 under RCP 8.5 scenario compared to 1970–2005

**Fig. 7:**
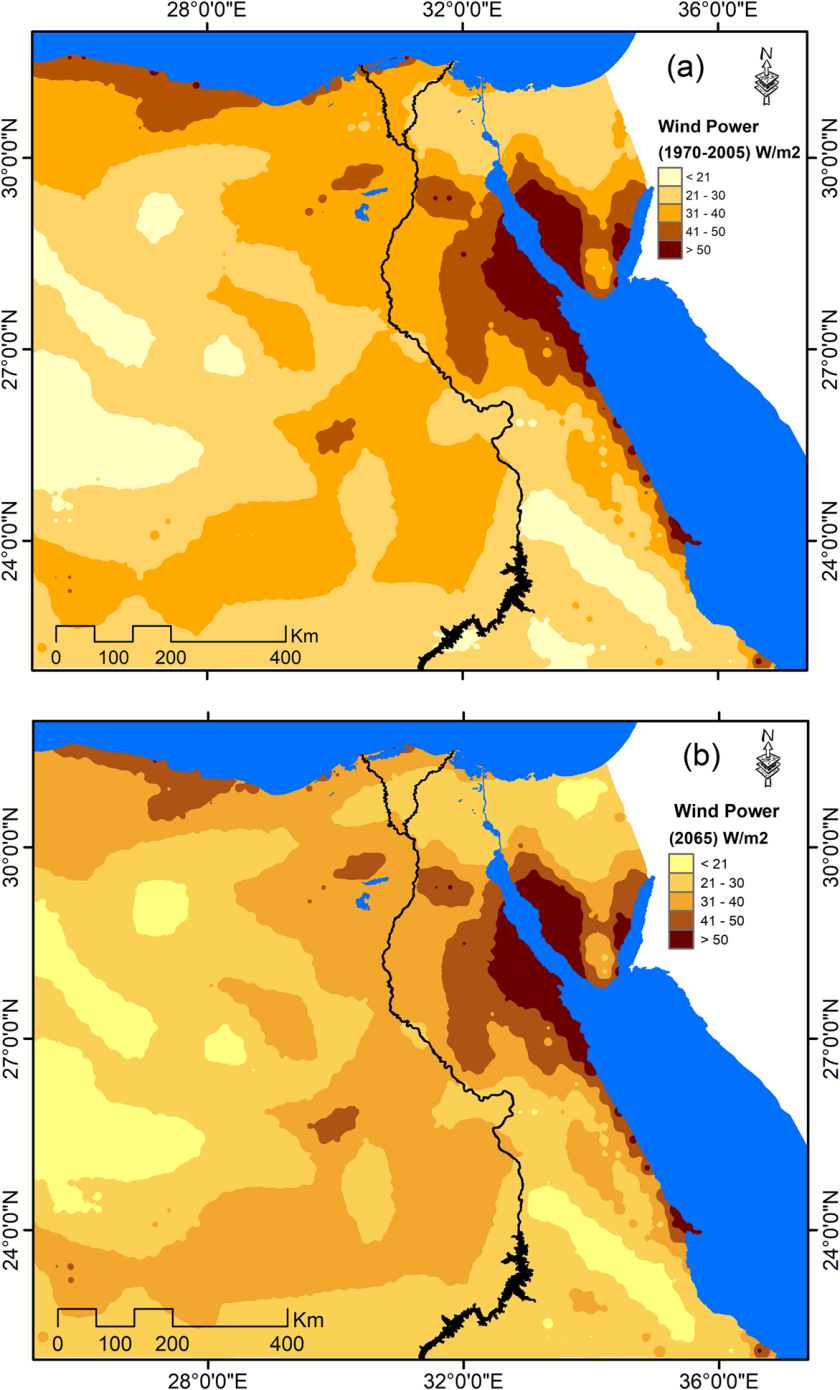
The available power in the wind: **a** 1970–2005 and **b** 2065 under RCP 8.5

Relatively, change rates of available power in wind in Egypt by the year 2065 are estimated to range between + 12% and − 12% (Fig. 6). Yet, absolute figures revealed that there is limited difference in available power in wind by the year 2065 under RCP 8.5 scenario that was estimated to range between 11.99 and 122.33 W/m^2^, compared to current levels, which was estimated to range between 12.34 and 112.7 W/m^2^ (Fig. 7).

Also, it was noticed that the available power in wind in those parts with a relatively high potential exceeding 40 W/m^2^, such as northwestern coastal zone, is expected to decrease by 38.1% by the year 2065. Similarly, these areas with highest potential for power generation above 50 W/m^2^ in the Suze Gulf are expected to decrease by 2.5% by 2065 under RCP 8.5.

Generally, such a relatively marginal projected change in wind speed and reduced extension of areas with high power available in wind may imply the limited potentials of wind power generation in the northern parts of Egypt. Such adverse impact of climate change on the future of wind power in Egypt may impede strategies for wind power generation expansion as one of renewable energy sources and necessitates the need for adopting other renewable sources than wind.

### Change in solar power

Currently, the maximum output power of commonly used PV modules in the market under standard testing conditions ranges between 320 and 450 W. Under temperature conditions of the baseline climate period (1970–200), the output PV module power was estimated to range between 301 and 340 W with an average of 310 W.

**Fig. 8:**
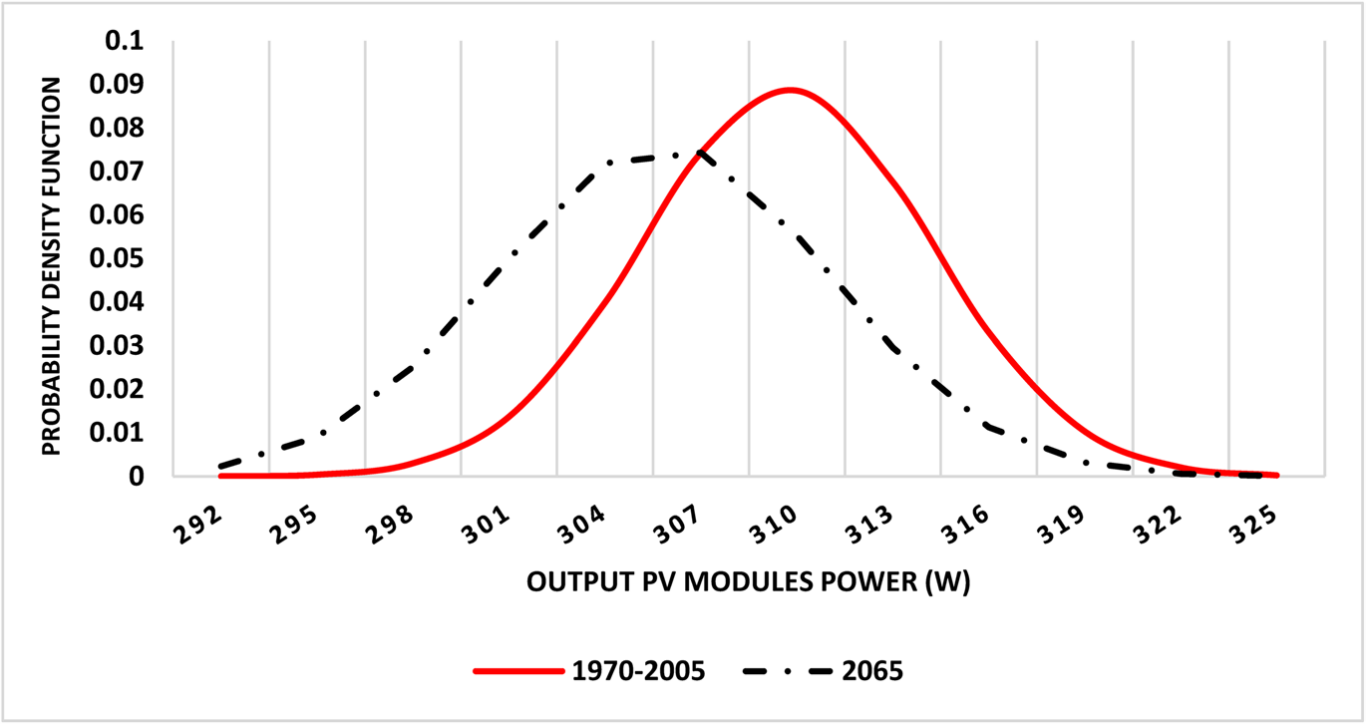
Gaussian distribution of projected output PV module power by the year 2065 under RCP 8.5 scenario compared to 1970–2005

Under RCP 8.5 scenario, different parts of Egypt are expected to experience increase in annual average temperature ranging between 2.3 and 3.4 °C with an average of about 3 °C by the year 2065. As a result of such temperature increase, the efficiency of solar panel is expected to be reduced. Accordingly, the projected power of PV modules is expected to decline to reach about 308 W on average (Fig. 8).

**Fig. 9:**
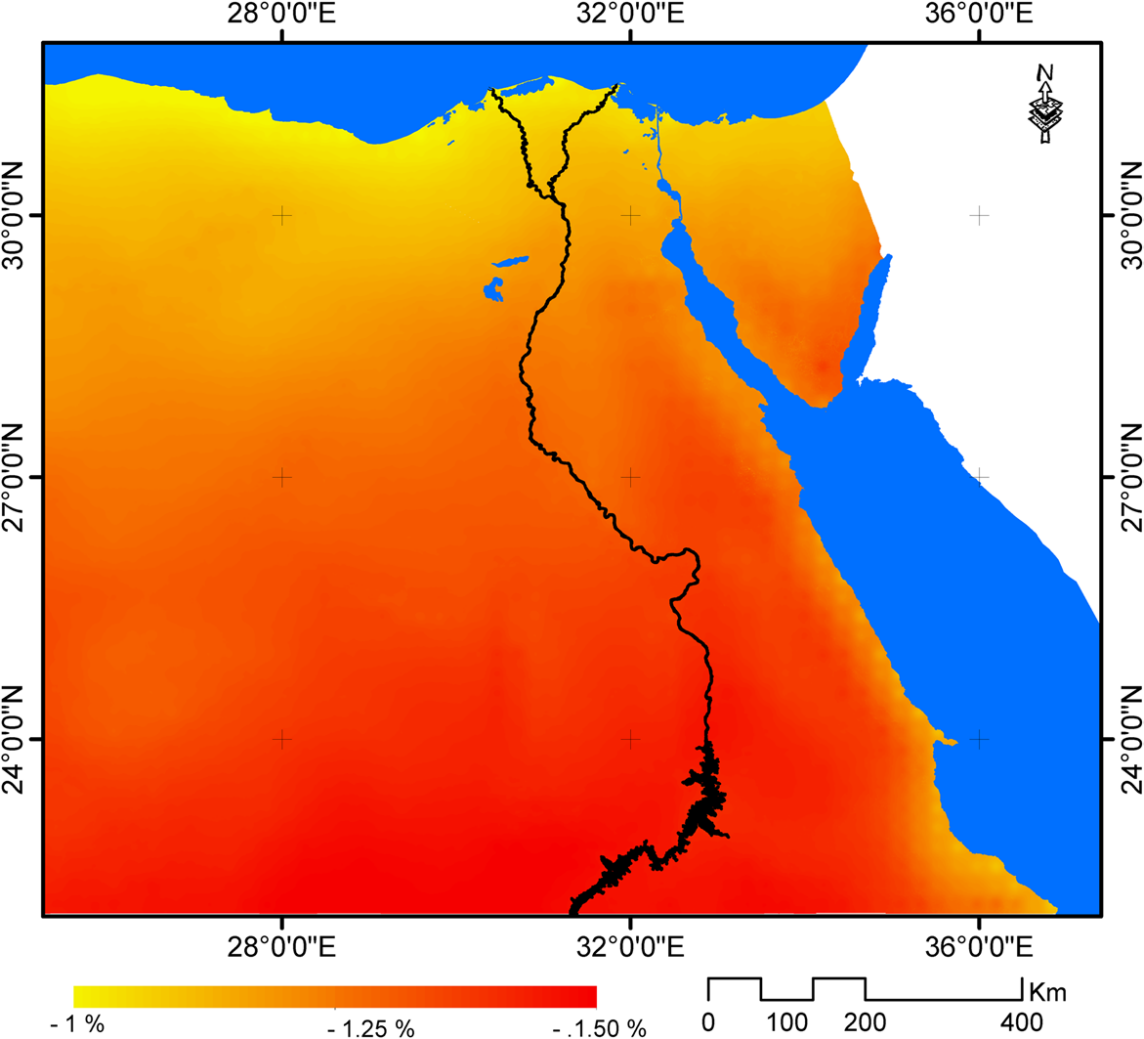
Relative change in output PV module power by the year 2065 under RCP 8.5 scenario compared to 1970–2005

This means that climate change and associated temperature increase are expected to have slight effect on PV module power. In this respect, it was found that the change in PV module power, as a result of temperature increase, is estimated to range between − 0.9 and − 1.5% with an average of − 1.3% compared to PV module power under current temperature (Fig. 9).

Also, it was noticed that that the highest reduction in PV module power will be in upper Egypt that are expected to experience the highest temperature increase and consequently considerable decline in solar energy generation potential. Such expected reduction in PV module power due to temperature increase may hinder the potential of solar energy in upper Egypt that receives usually the highest annual solar irradiation that is estimated to be about 2500 kWh/m^2^. At the same time, the northern parts of Egypt are expected to have the highest solar power potential as a result of the synergistic effect of both solar irradiation values and relatively low temperatures increase.

It should be noted that the magnitude and pattern of climate change impacts on wind and solar power in Egypt depend on several assumptions including no drastic change in available wind and solar power technologies, and business-as-usual emission at global level will lead to climate change scenario RCP8.5. Additionally, the uncertainty of the results is subject to the uncertainties of climate change scenario employed.

## Conclusion

Renewable energy is expected not only to affect climate change but also to be affected by climate change projected trends. The paper in hand aimed to assess the potential impacts of climate change on renewable energy sources in the case of Egypt. It was found, in this respect, that different parts of Egypt are expected to experience varied trends of changes in the annual wind speed, relative to 1970–2005. For example, while the wind speed is expected to decrease in the northern coastal zone, it is expected to increase in other parts of Egypt under RCP 8.5 scenario by the year 2065 compared to baseline climate period. Yet, the relative change in wind speed is expected to be marginal recording ± 5%. As a result of such expected marginal changes, the change in wind energy potential in different parts of Egypt by the year 2065 under RCP 8.5 scenario was estimated to range ± 12% compared to baseline climate period (1970–2005). This may indicate the limited potential of wind energy in Egypt under climate change.

Meanwhile, climate change is expected to have slight impact on solar power potential in Egypt through reducing PV module power by about 1.3% on average. It should be noted that the northern parts of Egypt will have a relatively higher solar energy potential compared to upper Egypt under RCP 8.5 scenario compared to reference period (1970–2005). Accordingly, such varied impacts of climate change on wind and solar energy should be carefully considered in strategic planning for renewable energy in Egypt. For example, future policy for renewable energy should focus on solar energy that is slightly affected by climate change compared to wind energy.

## Data Availability

The datasets generated during and/or analyzed during the current study are available from the corresponding author on reasonable request.
